# High Dose Antipsychotic Prescribing and Monitoring in Patients With Severe Mental Illness, in a Community Mental Health Service

**DOI:** 10.1192/bjo.2026.11814

**Published:** 2026-06-30

**Authors:** Samuel Oranu

**Affiliations:** North Staffordshire Combined Healthcare NHS Trust, Stoke on Trent, United Kingdom

## Abstract

**Aims:**

Assessing compliance with guidance on high-dose antipsychotic treatment (HDAT) prescribing and monitoring in patients with severe mental illness (SMI) managed within a community mental health service. This includes identification and documentation of HDAT prescribing, completion of HDAT monitoring forms, and adherence to recommended baseline, early, and longitudinal physical health monitoring.

**Methods:**

A retrospective audit was conducted of all patients under the SMI pathway (n= 391), in an outpatient clinic in North Staffordshire Combined Healthcare NHS Trust, who were prescribed high-dose antipsychotics over a 12-month period (January–December 2023). Data were extracted from electronic patient records. An electronic data collection tool,adapted from the Trust’s Standard Operating Procedures for HDAT prescribing and monitoring, was used. Collected data included documentation of HDAT status, completion of HDAT monitoring forms, and evidence of recommended investigations at the initiation phase (<12months) and continuation phase (>12 months) as well as side-effect monitoring using the Glasgow Antipsychotic Side-effect Scale (GASS). Compliance was assessed against Trust standards.

**Results:**

Of the 391 patients under the SMI pathway, 13 (3.3%) were prescribed high-dose antipsychotic treatment (HDAT). All patients on HDAT had a diagnosis of schizophrenia and were aged 49 years or older. Combination antipsychotic therapy accounted for 77% of HDAT prescribing, with 30% including a depot preparation. Olanzapine was the most frequently prescribed antipsychotic (n=6) and was associated with the highest dose exposure, reaching 150% of the BNF-licensed maximum dose for age and indication.

One patient was in the initiation phase of HDAT and had appropriate baseline investigations completed, including HDAT documentation, blood tests, ECG and physical observations: but not 3-monthly monitoring. The remaining 12 patients were in the continuation phase, of whom 91.7% had been identified. None, however, had completed HDAT monitoring forms or 3-monthly monitoring updates. While all patients received annual blood tests, ECGs and GASS assessments, 6-monthly monitoring was lacking. Factors contributing to incomplete documentation included inadequate identification of newly transferred patients already on HDAT, and record sharing barriers between GP’s and CMHS.

**Conclusion:**

HDAT prescribing was uncommon, but its use was predominantly identified retrospectively, rather than clinical decisions to commence HDAT. These findings highlight gaps in systematic identification, documentation, and ongoing monitoring, underscoring the need for improved integrated care, clear shared responsibility, and adherence to HDAT monitoring protocols to optimise patient safety.

